# Extracting Teeth

**Published:** 1865-12

**Authors:** 


### EXTRACTING TEETH.

In the whole range of surgery, no other operation is required so frequently as the extraction of teeth. In few, or none, is there a greater demand for common sense, on the part of the operator. The amputation of an arm, or a leg, is an operation less complex than the extraction of a tooth. One amputation is very much like another; but in extracting, there is an endless variety. What instrument is to be used, how it is to be applied, the amount and the direction of the force, have each to be decided, for every individual case.

But, notwithstanding all this, the public mind looks upon the operation as a trifling one; and too many in the profession sympathize with the sentiment. The public feeling is, "that anybody can pull a tooth;" and in the profession, many feel so indifferent, that they neglect to supply themselves with a complete set of instruments. I have often met professional brethren, who are quite careful about their excavators and pluggers, while very careless as to both quality and quantity of extracting instruments. This is inexcusable; indeed, reprehensible; for, as extraction is the most painful operation the dental surgeon is called on to inflict, he should be especially careful in regard to it. He should thoroughly understand every principle involved in the operation, and should possess every instrument that may be needed, and should see that it is always in perfect condition. And in the manipulations necessary, every motion should be firm, deliberate, and completely under the control of the will. The observing faculties should have the fullest play. In short, the operator should be thoroughly self possessed. But all this may appear to the reader as "trifling truisms, clothed in great swelling words of vanity;" but it is needed, nevertheless. I have seen experienced operators shut their eyes during the operation, their own faces distorted worse than those of their patients, and their countenances indicating anything but attention to that in which they were engaged.

Some in our profession speak as if it were disreputable to perform it. If a man extracts a good many teeth, they regard him as a mutilator — a butcher. But the truth may be that he is well provided with instruments, is attentive and kind, and, consequently, inflicts less pain than his neighbors. At the late meeting at Chicago, some one spoke of having extracted fifty teeth in a day. It would have been nearly as much to his credit, in that crowd, to have confessed himself a pickpocket. It was taken for granted, that he had destroyed many teeth which might have been saved. But this may not be true. Such wholesale destruction is a thing quite too melancholy to boast of; and yet it is sometimes necessary. I once extracted fifty-two teeth in less than half a day, not one of which could have been saved by any skill of man. Many cases present themselves, for which nothing but extraction will answer. This is a sadly solemn truth; but it is a truth.

The loss of a tooth is a serious misfortune. The man who extracts one, without just cause, deserves to be punished. "A tooth for a tooth," is the gospel adapted to his case.

Let us labor to elevate the public and the profession, that the laws of health may be so observed, that mutilations will be less called for. W.

